# Do estimates of cost-utility based on the EQ-5D differ from those based on the mapping of utility scores?

**DOI:** 10.1186/1477-7525-6-51

**Published:** 2008-07-14

**Authors:** Garry R Barton, Tracey H Sach, Claire Jenkinson, Anthony J Avery, Michael Doherty, Kenneth R Muir

**Affiliations:** 1 Health Economics Group, School of Medicine, Health Policy and Practice, University of East Anglia, Norwich, UK; 2 School of Chemical Sciences and Pharmacy, University of East Anglia, Norwich, UK; 3 School of Community Health Sciences, University of Nottingham, Nottingham, UK; 4 Academic Rheumatology, University of Nottingham, Nottingham, UK

## Abstract

**Background:**

Mapping has been used to convert scores from condition-specific measures into utility scores, and to produce estimates of cost-effectiveness. We sought to compare the QALY gains, and incremental cost per QALY estimates, predicted on the basis of mapping to those based on actual EQ-5D scores.

**Methods:**

In order to compare 4 different interventions 389 individuals were asked to complete both the EQ-5D and the Western Ontartio and McMaster Universities Osteoarthritis Index (WOMAC) at baseline, 6, 12, and 24 months post-intervention. Using baseline data various mapping models were developed, where WOMAC scores were used to predict the EQ-5D scores. The performance of these models was tested by predicting the EQ-5D post-intervention scores. The preferred model (that with the lowest mean absolute error (MAE)) was used to predict the EQ-5D scores, at all time points, for individuals who had complete WOMAC and EQ-5D data. The mean QALY gain associated with each intervention was calculated, using both actual and predicted EQ-5D scores. These QALY gains, along with previously estimated changes in cost, were also used to estimate the actual and predicted incremental cost per QALY associated with each of the four interventions.

**Results:**

The EQ-5D and the WOMAC were completed at baseline by 348 individuals, and at all time points by 259 individuals. The MAE in the preferred model was 0.129, and the mean QALY gains for each of the four interventions was predicted to be 0.006, 0.058, 0.058, and 0.136 respectively, compared to the actual mean QALY gains of 0.087, 0.081, 0.120, and 0.149. The most effective intervention was estimated to be associated with an incremental cost per QALY of £6,068, according to our preferred model, compared to £13,154 when actual data was used.

**Conclusion:**

We found that actual QALY gains, and incremental cost per QALY estimates, differed from those predicted on the basis of mapping. This suggests that though mapping may be of value in predicting the cost-effectiveness of interventions which have not been evaluated using a utility measure, future studies should be encouraged to include a method of actual utility measurement.

**Trial registration:**

Current Controlled Trials ISRCTN93206785

## Background

Given that health care resources are scarce there is a need to evaluate the cost-effectiveness of different health care interventions. Within such studies economists generally seek to measure the benefits in terms of utility, a scale where 0 represents death and 1 is equivalent to full health, in order for the benefits of many interventions to be compared on a common scale [1-3]. However, as not all studies choose to measure outcomes in terms of utility, an increasing amount of research has now been conducted on mapping, where scores from a condition-specific (non preference-based) measure are 'converted' into a utility (preference-based) score using a pre-defined formulae [4]. Mapping thereby presents the possibility of estimating the cost-utility (i.e. the incremental cost per quality adjusted life year (QALY) [1]) of interventions that have previously only been evaluated using a condition-specific measure. Indeed a number of mapping models have now been developed [5-19], the use of mapping has been considered by the UK National Institute of Health and Clinical Excellence (NICE) [20], and mapping has been used to estimate the utility scores, and in turn cost-effectiveness, of a number of health care interventions [21-23]. The role of this paper is to assess the criterion validity of such mapping procedures, as this represents an area where little research has been undertaken. We achieve this by comparing the actual estimated QALY gain associated with different interventions to the QALY gains predicted on the basis of mapping models, and similarly comparing the actual incremental cost per QALY estimates associated with those interventions to those derived from mapping models.

## Methods

### Participants

All individuals were taking part in the Lifestyle Interventions for Knee Pain (LIKP) study, which was designed to compare the effectiveness and cost-effectiveness of four different interventions. The four interventions were receipt of a leaflet, advice on knee strengthening exercises, dietary advice, and both dietary and exercise advice (hereafter these interventions are referred to as 1, 2, 3 and 4 as the main focus of this paper is methodological). Ethical approval for this study was granted by the UK Nottingham Research Ethics Committee. Recruitment into the LIKP study began in May 2003 and ended in March 2005, where all registered patients in five Nottingham general practices who were aged ≥ 45 years, and deemed (by their general practitioner) to be well enough to complete a questionnaire, were sent an ascertainment questionnaire. Additionally a local media campaign was conducted, which included adverts in the local press and on the local radio. Responding individuals were recruited into the LIKP study if they reported that they had had knee pain on most days of the last month, were aged ≥ 45 years, had a body mass index (BMI) > 28.0 kg/m^2^, and gave consent to be randomised to one of the four interventions.

#### Outcome measures

At both pre-intervention (baseline) and post-intervention (at 6, 12 and 24 months) participants in the LIKP study were asked to complete both the WOMAC (Western Ontario and McMaster Universities Osteoarthritis Index) and the EQ-5D.

The WOMAC contains 24 questions and measures the amount of pain (5 questions), stiffness (2 questions), and difficulty in physical functioning (17 questions), where the response options are none (0), mild (1), moderate (2), severe (3) or extreme (4) [24]. Scores can thereby range between 0 and 20 on the pain sub-scale (pain), 0 and 8 on the stiffness sub-scale (stiffness), 0 and 68 on the functioning sub-scale (functioning), and sum to between 0 to 96 (total WOMAC), where higher scores denote a worse response [25,26]. Previous evidence of the adequate performance of the WOMAC has been shown for construct validity [27] and responsiveness [28,29].

The EQ-5D has five questions, where the respondent is asked to report the level of problems they have (no problems, some/moderate problems, and severe/extreme problems) with regard to mobility, self-care, usual activities, pain/discomfort, and anxiety/depression [30]. Responses to these five dimensions are converted into one of 243 different EQ-5D health state descriptions, which range between no problems on all five dimensions (11111) and severe/extreme problems on all five dimensions (33333). A utility score was assigned to each of these 243 health states using the York A1 tariff [31], which was based on the preferences elicited from a survey of 3395 UK residents – EQ-5D scores range between -0.594 and 1 (full health).

### Statistical analyses

#### Overview

We adopted a split-sample approach to the mapping of condition-specific scores into utility scores. The baseline scores from the aforementioned LIKP study were used to develop various mapping models (to predict the EQ-5D scores). The performance of those models was then assessed on the post-intervention scores in order to identify our preferred model. Finally, for each of the four interventions, the actual QALY gains (over the 24 month trial period), and the incremental cost per QALY estimates, were compared to those that would have been predicted on the basis of our preferred model.

#### Model specification

In line with previous mapping models [9,11-14,16-19,22,23], we used linear regression analysis to predict the relationship between scores on a condition-specific measure and scores on a utility measure. Using baseline WOMAC and EQ-5D data from the LIKP study, five models were developed, starting with the most parsimonious. In each of the models different baseline WOMAC scores took the form of independent variables and the baseline EQ-5D score acted as the dependent variable. The predictor variables in each of the five models were as follows.

Model A: total WOMAC;

Model B: pain, stiffness, functioning;

Model C: total WOMAC, total WOMAC^2^

Model D: pain, stiffness, functioning, pain*stiffness, pain*functioning, stiffness*functioning, pain^2^, stiffness^2^, functioning^2^;

Model E: best of above models plus patient characteristics of age and sex.

#### Model performance

We sought to identify our 'preferred' model, out of the five aforementioned models, by comparing actual EQ-5D scores to EQ-5D scores predicted on the basis of each of the five mapping models. This comparison was performed at 6, 12, and 24 months post-intervention for individuals who had complete study data (i.e. completed both the EQ-5D and each of the WOMAC sub-scales at all of the four time points within our study). Baseline data was not used within these comparisons as, in line with previous studies [8,19], we sought to assess the performance of the models on a different sample of data to that used to develop the models. We inferred the 'preferred' model to be the one with the lowest Mean Absolute Error (MAE), where the MAE was calculated by taking the average value of each absolute prediction error (the prediction error equals the difference between the actual EQ-5D score, for a particular individual, and, for the same individual, the EQ-5D score predicted on the basis of the mapping model). For each model we also report the adjusted r^2^squared and the root mean square error (RMSE) (the RMSE is the positive square root of the average squared prediction error). Finally, in order to assess how well the mapping formulae performs across the range of EQ-5D scores, we also plot the actual follow-up EQ-5D scores against the prediction errors (predicted score minus actual score) from our inferred preferred model.

In line with the mapping models which are developed here, a previous study has also attempted to predict utility scores using scores on the WOMAC [19]. The study differed from ours in that it measured utility using the Health Utilities Index [32], rather than the EQ-5D, and whilst acknowledging that there is an argument that utilities derived from different instruments should not be compared [33], we also sought to compare the utility scores predicted by the mapping models of Grootendorst et al. [19] to our actual EQ-5D scores. Grootendorst et al. [19] developed four models, but we were only able to predict utility scores using the coefficient values from two of their models (here referred to as G1 and G2) as the other two models used independent variables (e.g. duration of osteoarthritis) which were not available for the individuals within our study. Models G1 and G2 had the same independent variables as our Model D, and G2 also included the variables of age and sex. The performance of these two models was again assessed by calculating the MAE, RMSE and the adjusted r^2^squared. Additionally, we also identified our 'preferred' Grootendorst et al. model to be that which had the lowest MAE.

### Comparing actual trial results to those predicted using mapping models

#### QALY gain

The LIKP study sought to estimate the effectiveness of four different interventions. We thereby used the following methods to compare the mean QALY gain (measured from baseline over the 24 month trial period) for each of the four interventions, as estimated by actual data, to that predicted on the basis of our preferred mapping model and our preferred Grootendorst et al. model. With regard to actual data, for those participants who had complete study data, the baseline and post-intervention (6, 12 and 24 month) EQ-5D scores were used to estimate the QALY gain using the area under the curve (AUC) method, with adjustment for baseline scores [34]. The mean QALY gains were then calculated by estimating the average QALY gain for the four groups of participants who received each of the four interventions.

With regard to our preferred model, and our preferred Grootendorst et al. model, EQ-5D scores were predicted at baseline, 6, 12, and 24 months post-intervention for individuals who had complete study data (EQ-5D scores were predicted at all time points as this method would be used for studies which had not included a measure of utility). Using the same methods as for the actual EQ-5D data (see above), these predicted EQ-5D scores were then used to estimate the QALY gain for each individual who had complete study data, and the mean QALY gain for each intervention, where these calculations were performed for both our preferred model, and our preferred Grootendorst et al. model. Finally, we compared the mean QALY gain for each of the four interventions according to actual EQ-5D data to that predicted on the basis of our preferred mapping model, and our preferred Grootendorst et al. model. The paired t-test was also used to assess whether the actual mean QALY gains differed significantly (p < 0.05) from those predicted on the basis of our preferred model and our preferred Grootendorst et al. model. Finally, it should be noted that discounting was not undertaken in any of the above analysis as we sought to identify the differences between the QALY gains based on actual EQ-5D scores and those based on predicted EQ-5D scores, and we sought to identify the difference that arose due to the use of the mapping procedure.

### Incremental cost per QALY

As described elsewhere (Barton GR, Sach TH, Avery AJ, Doherty M, Jenkinson C, Muir KR. Lifestyle Interventions for Knee Pain: Cost-effectiveness analysis. Paper submitted for publication), levels of resource use were combined with unit cost data to estimate the change in cost over the two year study period for all participants in the LIKP study (costs were calculated at 2005/6 levels, but in order to ensure the same discount rate was applied to both costs and benefits future costs were not discounted). The change in costs, rather than total costs, was estimated due to the presence of baseline differences in the analgesic costs across each of the four interventions, and was calculated using the same aforementioned AUC technique as was undertaken for QALYs. The mean change in cost was thereby calculated for each of the four interventions. These values along with the previously calculated QALY gains based on i) actual EQ-5D scores, ii) the EQ-5D scores predicted from our preferred model, and iii) the EQ-5D scores predicted by our preferred Grootendorst et al. model were then used to estimate the incremental cost per QALY gain associated with each of the four interventions (this is commonly referred to as the incremental cost-effectiveness ratio (ICER) [1], and hereafter we refer to it as the incremental cost per QALY). Incremental cost per QALY estimates were made by ordering the four interventions from least costly to most costly, excluding those interventions which were dominated (had a higher mean change in cost and lower mean QALY gain than another intervention) or were subject to extended dominance (combinations of other interventions could provide a higher benefit at lower/equivalent cost), and then calculating the ICER (incremental cost/incremental effect) for remaining interventions. Separate incremental cost per QALY estimates were made using each of the aforementioned three different methods of calculating a QALY gain. Finally, it should be noted that all analyses were performed in either SPSS [35] or Microsoft Excel.

## Results

### Participants

Across the five general practices 12,500 individuals were sent an ascertainment questionnaire, and 8,044 (64.4%) were returned. Subsequently, 318 individuals met the entry criteria for the LIKP study, and gave consent to be randomised to one of the four interventions. An additional 71 individuals were recruited via the media campaign. The mean age of these 389 individuals was 62.0 years, 66.0% were female, and 23.4% were classified as overweight (BMI 25 to < 30 kg/m^2^), 50.4% as class I obese (30 to < 35 kg/m^2^), 16.9% as class II obese (35 to < 40 kg/m^2^), and 9.9% as class III obese (≥ 40 kg/m^2^). At baseline 348 individuals fully completed both the EQ-5D and the WOMAC, and data for these individuals were used to develop the five mapping models. The mean score (95% confidence interval) for these 348 individuals was 0.557 (0.528 to 0.587) on the EQ-5D, 7.76 (7.39 to 8.13) on the pain sub-scale, 3.91 (3.74 to 4.07) on the stiffness scale, 27.89 (26.54 to 29.23) on the physical functioning scale, and 39.55 (37.77 to 41.34) on the total WOMAC scale.

### Statistical analyses

#### Model Specification and performance

**Figure 1:**
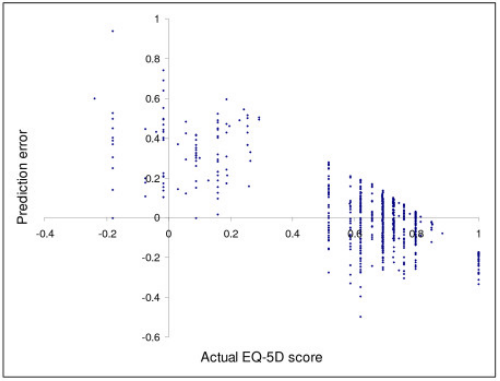
Comparison of the actual EQ-5D scores and the prediction errors of Model E.

The parameter estimates for each of the five models that we developed to predict the baseline EQ-5D scores are summarised in Table 1, where it should be remembered that a higher WOMAC score denotes a worse response. When these models were used to predict the EQ-5D scores for the 259 individuals who had complete study data, it can be seen that Model C had the lowest MAE (0.140) out of the first four models when the actual scores at 6, 12 and 24 months were compared to those predicted on the basis of these models. As such, Model E used the same independent variables as model C, with the additional variables of age and sex. Model E had an MAE of 0.129, and was thus deemed to be our preferred model (see Appendix 1 for full details of both Models C and E). By way of an example of how these models are used to estimate EQ-5D scores, our preferred model would predict that a male with the aforementioned mean baseline characteristics (age = 62 years; total WOMAC = 39.55) would have an EQ-5D score of 0.577 (-0.3474012785 + (-0.0005977709*39.55) + (-0.0001081560*39.55^2^) + (0.0326027536*62) + (-0.0002352456*62^2^) + (0.0475889687*0)), the actual mean baseline EQ-5D score was 0.566 (95% confidence interval 0.532 to 0.600). Figure 1 shows how the prediction errors (predicted score minus absolute score) of our preferred model (E) vary according to the actual EQ-5D scores (6, 12 and 24 month post-intervention data are plotted).

**Table 1:** Parameter estimates for the five models which were used to predict the baseline EQ-5D scores.

	Model				
	A	B	C	D	E
Intercept	0.900‡	0.886‡	0.747‡	0.691‡	-0.347
total WOMAC	-0.009‡		0.001		-0.001
pain		-0.012		-0.009	
stiffness		0.006		0.092†	
functioning		-0.009‡		-0.005	
pain* stiffness				-0.005	
pain*functioning				-0.001	
stiffness*functioning				0.001	
pain^2^				0.002	
stiffness^2^				-0.011	
functioning^2^				0.000	
total WOMAC^2^			-0.000‡		-0.000†
age					0.033*
age^2^					0.000
sex (if Female)					0.048
Range of predicted scores	0.086 to 0.900	0.086 to 0.900	-0.235 to 0.748	-0.111 to 0.852	-0.184 to 0.828
MAE	0.148	0.147	0.140	0.146	0.129
Adjusted r^2^	0.275	0.274	0.296	0.299	0.313
RMSE	0.189	0.187	0.185	0.190	0.180

* = p < 0.05, † = p < 0.01, ‡ = p < 0.001, MAE = Mean Absolute Error, RMSE = Root Mean Squared Error. See Appendix 1 for full details of Model C and Model E.

The parameter estimates for models G1 and G2 are published in the appendix of the paper by Grootendorst et al. [19]. In terms of performance, model G1 had a lower MAE (0.142) than model G2 (0.144) and though both these MAE were higher than that for Model E they were lower than that for Model D, which used the same WOMAC predictor variables.

### Comparing actual trial results to those predicted using mapping models

#### QALY gain

The WOMAC and EQ-5D were fully completed at baseline, 6, 12 and 24 months post-intervention by 259 individuals (66.6% of trial participants). Based on the actual EQ-5D scores for these individuals the mean QALY gain (over the 2 year trial period, with adjustment for baseline scores), for each of the four interventions, was estimated to be 0.089, 0.081, 0.120 and 0.149, respectively. In Table 2 these values are compared to the mean QALY gains predicted by our preferred model (E), and our preferred Grootendorst et al. model (G1). It can be seen that, for each of the four interventions, the mean QALY gains derived from both these preferred models were consistently lower than the actual estimated mean QALY gains, though the results were not significantly different.

**Table 2:** Mean estimated QALY gains based on both actual data and mapping models.

Intervention	1	2	3	4
Actual results	0.089 (0.330)	0.081 (0.428)	0.120 (0.450)	0.149 (0.352)
Model E	0.006 (0.162)	0.058 (0.186)	0.058 (0.158)	0.136 (0.209)
Model G1	-0.009* (0.146)	0.027 (0.166)	0.062 (0.149)	0.114 (0.186)

Standard deviations are presented in brackets, * = p < 0.05 according to the paired t-test

### Incremental cost per QALY

Based on the responses for the 259 individuals who had complete study data the mean change in costs (standard deviation) for each of the four interventions (1–4) was estimated to be £7.75 (£122.66), £321.12 (£131.12), £832.85 (£171.39) and £792.24 (£248.19), respectively. These four mean change in costs were subsequently combined with the estimated mean QALY gains, based on both actual EQ-5D data and our preferred mapping models (as shown in Table 2), to give the incremental cost per QALY estimates which are reported in Table 3. Based on the actual EQ-5D scores intervention 4 had both a higher mean effect, and lower mean increase in cost, than intervention 3 – intervention 4 thereby dominated intervention 3, and for similar reasons intervention 1 dominated intervention 2. The incremental cost per QALY (ICER) for intervention 4 was thereby calculated by comparing it to intervention 1, and was estimated to be £13,154 ((792.24-7.72)/(0.149-0.089)).

**Table 3:** Estimated incremental cost per QALY based on both actual data and mapping models.

Intervention	1	2	3	4
Actual results	N/A	D by 1	D by 4	£13,154
Model E	N/A	£6,036	D by 4	£6,086
Model G1	N/A	Subject to ED	D by 4	£6,345

N/A = Not applicable (this intervention is the least costly and least effective), ED = Extended dominance, D = Dominated

These results, differed from those that were obtained when the EQ-5D scores were predicted on the basis of our preferred model, where though intervention 3 was still dominated by intervention 4 intervention 2 was no longer dominated by intervention 1. Instead intervention 2 had an ICER of £6,036 (when compared to intervention 1), and the ICER for intervention 4 was estimated to be £6,068 (when compared to intervention 2) (see Table 3). Similarly, though intervention 3 was still estimated to be dominated by intervention 4 when model G1 was used to predict the EQ-5D scores the incremental cost per QALY results were again different from those based on the actual EQ-5D scores: intervention 2 was now estimated to be subject to extended dominance as combinations of interventions 1 and 4 could provide a higher benefit at lower cost (see [36] for further information on how to determine when an intervention is subject to extended dominance), and the ICER for intervention 4 was estimated to be £6,345 (when compared to intervention 1) (see Table 3).

## Discussion

Within this paper we have shown how mapping models can be used to predict the QALY gain associated with different interventions, and in turn calculate the ICER associated with different interventions. When these predicted results are compared to actual results we found that our preferred model consistently underestimated the mean QALY gain associated with the four compared interventions. The ICER of each of the four interventions, based on actual data, also differed from that based on our preferred mapping model (see Table 3) – the most effective intervention (intervention 4) was estimated to be more cost-effective according to our preferred mapping model (ICER = £6,068), compared to when actual data was used (ICER = £13,154).

Within this paper we also calculated the incremental cost per QALY estimates for the four interventions in the LIKP study using a previously published mapping model based on the HUI3. This approach is justified by the following example. In a previous study by Thomas et al. [37] it was found that a home exercise programme for people with knee pain was more effective than no intervention, and more costly, but as effectiveness was only measured on the WOMAC one could not compare the cost-effectiveness of this new intervention to other health-care interventions, or the cost-effectiveness threshold. One possible way of estimating the cost-effectiveness of the home exercise programme would be to convert the WOMAC scores into utility scores using the mapping scores published by Grootendorst et al. [19]. Interestingly, within this paper, our preferred Grootendorst et al. model had a lower MAE than was the case in the original data set within which it was developed (0.142 compared to 0.1645). Furthermore, the predicted incremental cost per QALY estimates based on our preferred Grootendorst et al. model, which was developed using the HUI3, were also numerically closer to the actual incremental cost per QALY estimates, than was the case for the predicted cost per QALY estimates based on our preferred model, which was developed using the EQ-5D (see Table 3).

### Explanations

**Figure 2:**
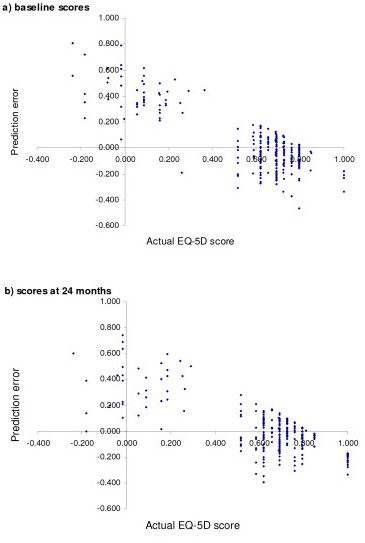
Comparison of the actual EQ-5D scores and the prediction errors of Model E.

One possible explanation for the above QALY differences is as follows. Figure 1 shows that that the prediction errors of our preferred model tend to be increasingly positive for lower EQ-5D scores and increasingly negative for higher EQ-5D scores. This suggests that the regression would tend to over predict the EQ-5D score for those at low levels of utility, and under predict the EQ-5D score for those at high levels of utility. As the EQ-5D scores tend to increase post-intervention (baseline mean EQ-5D score = 0.566 and 24 month mean = 0.639, for N = 259), the consequence of this is that the final EQ-5D scores tend to be underestimated and thus the QALY gain associated with each of the four interventions also tends to be underestimated. This is further demonstrated by plotting the predicted errors against the actual EQ-5D scores at baseline (Figure 2a) and at 24 months post-intervention (Figure 2b), where the fact that the prediction errors are more likely to be negative at 24 months indicates that the actual scores were more likely to exceed the predicted scores at this time period, than was the case at baseline. However, it should be noted that, even though the effectiveness of each intervention was predicted to be lower by the mapping models, the most effective intervention (4) was estimated to have a more favourable incremental cost per QALY estimate according to the mapping models as the predicted mean QALY gain for this intervention was closer to the actual estimate, than was the case for the three other interventions (see Table 2).

A further explanation as to why the QALY gains predicted by the mapping formulae tend to underestimate the actual QALY gain may be that the benefits of the interventions are not fully captured by the WOMAC, which concentrates on pain, stiffness and physical functioning. Thus, if there are other benefits, which are not detected by the WOMAC, then this may also explain why the mapping formulae tends to under predict the actual QALY gains associated with each of the four interventions. This hypothesis concurs with that of others who have pointed out that mapping models can only encompass the gains that are detected by the condition-specific measure [10].

### Comparisons with other studies

We are aware of two papers which have compared both the QALY gain and incremental cost per QALY predicted on the basis of different mapping formulae [38,39]. Pickard et al. [38] used ten different mapping models, four of which were based on the SF-12 [40], and six on SF-36 [41]. They then used both the before and after scores from both a group of asthma patients, and a group of stroke patients, to estimate the 1 year QALY gain, and the associated incremental cost per QALY (for both groups of patients it was assumed that the incremental cost of the intervention was $2000 greater than standard treatment). They found that the QALY gain (incremental cost per QALY) estimates for the asthma patients ranged between 0.032 and 0.065 ($30,769 to $63,492), and that the QALY gain for the stroke patients ranged between 0.028 to 0.072 ($27,972 to $72,727) [38]. Thus, the results of Pickard et al. [38], are in line with ours, in that the estimated ICER varies according to which algorithm is used. This was also the case in the study by Marra et al. [39], who used mapping to estimate the ICER associated with two different drug strategies for patients with rheumatoid arthritis. Using a previous database, mapping models were created by estimating the relationship between the Health Assessment Questionnaire [42] and the utility measures of the EQ-5D [30], SF-6D [43], HUI2 and HUI3 [32]. The four created mapping models were then used to estimate the QALY gain associated with the two different drug strategies in a different data set, where the results for the two treatments were 3.33 and 4.67 for the EQ-5D mapping model, 3.79 to 4.69 for the SF-6D, 4.16 to 5.33 for the HUI2, and 1.73 to 3.68 for the HUI3. These values were used to estimate the incremental effect, and when accompanied by estimates of the incremental cost, the ICER was estimated to range between $32,018 (HUI3) and $69,826 (SF-6D).

We also sought to compare our results to those of others who have used similar techniques to estimate the relationship between scores on a condition-specific measure and scores on a utility measure [9,11-14,16-19,22,23]. Not all of these studies reported the mean absolute error (MAE) but reported values did include < 0.13 [17], 0.14 to 0.16 [17,18], 0.1628 [19] and 0.19 [12], all of which are generally comparable to the MAE of our preferred model (0.129). Within these other studies, and the mapping models that we developed (see Table 1), there was also a tendency for the agreement between the observed and predicted utility scores to improve as further socio-demographic variables were used to predict the variation in the utility scores (in an attempt to ensure that others can use the mapping formulae that we developed we only included the socio-demographic variables of age and sex in our preferred model as we considered that other variables would not be routinely recorded in other studies).

### Implications

One possible implication of the results presented here is that, as the scores predicted on the basis of mapping differ from actual scores, utility estimates that are based on mapping models should not be seen as a substitute for actual utility measurement. As a consequence, prospective clinical trials should seek to measure outcomes with a utility measure, rather than using a condition specific measure and a mapping model to estimate the utility gain associated with an intervention. There may however still be a role for mapping in terms of estimating the utility score in previous studies which have only included a non-preference based outcome measure. For example, in the aforementioned study by Thomas et al. [37], it was found that the new intervention was more costly and more effective (on the WOMAC), but without translating the scores on the WOMAC into utility scores we have no way of trying to estimate whether the additional benefits are worthwhile i.e. we can not determine whether it is cost-effective to provide the new intervention, or whether it would be more cost-effective to spend scarce health care resources elsewhere. Indeed a recent report by the UK NICE relied heavily on the use of mapping to estimate the cost-effectiveness of a number of interventions concerned with the care and management of osteoarthritis in adults [44]. Further justification for the use of mapping is also provided by the results of this study, in that had we only measured outcomes with the WOMAC, and used a previously published mapping model [19] to estimate the QALY gains, and incremental cost per QALY, associated with each of the four interventions (as we did in Table 3) then we would have come to the same conclusion i.e. that intervention 4 was the most cost-effective intervention as it has an incremental cost per QALY which is less than the £30,000 per QALY cut-off which has been argued to represent the approximate cost-effectiveness threshold which has been used by NICE [45-47] (NICE states that it operates to a threshold range of £20,000 to £30,000 per QALY [48]).

### Strengths and weaknesses

We consider the main potential limitation of our study to be that the results may not be generalizable. This arises because we only used the WOMAC to predict the EQ-5D score, and other studies which use a different condition-specific measure, or utility measure, may find the actual study results are more similar to those predicted on the basis of the mapping models. Our results are however important as evidence as to the validity of mapping, in terms of how closely incremental cost per QALY estimates based on actual results align to those based on mapping, can only be provided by a series of converging results [49]. Moreover, in an attempt to increase the generalizability of our results we sought to develop mapping formulae using the technique of linear regression as this is the technique that is most commonly used within the literature [9,11-14,16-19,22,23]. We do however appreciate that, as the utility scale is bounded, the technique of linear regression can result in biased and inconsistent estimates [50,51]. As such it may be that other techniques such as Tobit regression, the censored least absolute deviations (CLAD) estimator [9] and restricted maximum likelihood (REML) [8] could result in a smaller MAE and better prediction.

A further potential weakness of this paper is that we have used various approaches (e.g. a complete case analysis and discounting of future costs and benefits at 0%) which might not be undertaken in standard cost-effectiveness analysis. Techniques such as discounting and imputation [1] were not used as the main focus of this paper was methodological, and we sought to identify the true difference between actual and predicted scores that arose due to mapping. Additionally, we did not explore the potential for selection bias that might arise due to the recruitment of participants from two different sources (recruitment via local general practices compared to the local media campaign).

The main strength of our paper is that this is one of the first studies to compare the actual QALY gain associated with particular interventions to the QALY gain that would have been predicted, for the same interventions, on the basis of mapping. Moreover, this comparison has been undertaken using both mapping models developed from both the LIKP study data, and from a previous paper [19]. We are aware of two papers [38,39] who make similar incremental cost per QALY comparisons. We build upon the first of these papers [38] by making incremental cost per QALY comparisons using mapping models that are based on a condition-specific measure, rather than the more generic measures of the SF-12 [40] and SF-36 [41], by using actual cost estimates for actual interventions, and by making comparisons with a different measure of utility (the EQ-5D). We similarly advance upon the second paper [39] as they undertook incremental cost per QALY calculations using a number of mapping formulae, but did not include an actual measure of utility within their comparisons.

## Conclusion

We have shown how mapping can be used to estimate both the QALY gain, and incremental cost per QALY, associated with different interventions, and compared these predictions to actual results. In our study the mapping models developed from the WOMAC tended to underestimate the QALY gain associated with each of four interventions, compared to that which was derived from actual EQ-5D scores. Similarly, the incremental cost per QALY estimates based on the mapping models also differed from those based on actual data. This suggests that future trials should include a measure of utility, however mapping may still be useful in estimating the cost-effectiveness of interventions which have previously only been evaluated with a condition-specific measure.

## Appendix 1

Model C: Predicted EQ-5D score = 0.746652555353163 + (0.000810215321934668* total WOMAC) + (-0.000119664323424435* total WOMAC^2^)

Model E: Predicted EQ-5D score = -0.3474012785 + (-0.0005977709* total WOMAC) + (-0.0001081560* total WOMAC^2^) + (0.0326027536*age) + (-0.0002352456*age^2^) + (0.0475889687*sex))

sex is equal to 1 if Female and 0 if male.

## Competing interests

The authors declare that they have no competing interests.

## Authors' contributions

GB and TS conceived the idea for the paper, undertook the analysis and drafted the paper. CJ, AA, MD, and KM assisted in the acquisition of data, interpretation of the analysis, and commented on drafts of the manuscript. All authors read and approved the final manuscript.
